# Crystal structure and doping in synthetic enstatite: an analysis of Li/Fe^3+^-doped single-crystal samples

**DOI:** 10.1107/S2052520624011624

**Published:** 2025-01-14

**Authors:** Paolo Ballirano, Beatrice Celata, Alessandro Pacella, Andrea Bloise, Ferdinando Bosi

**Affiliations:** a Department of Earth Sciences, Sapienza University of Rome, Piazzale Aldo Moro 5, I-00185, Rome, Italy; b Department of Energy Technologies and Renewable Sources, ENEA CR Casaccia, S. Maria di Galeria, 00123, Rome, Italy; c Department of Biology, Ecology and Earth Science, University of Calabria, Via P. Bucci cubo 15b, I-87036, Arcavacata di Rende (CS), Italy; University of Geneva, Switzerland

**Keywords:** SCXRD, SREF, Mg_2_Si_2_O_6_, LiFe^3+^Si_2_O_6_, py­rox­enes, orthoenstatite, protoenstatite

## Abstract

A series of Li^+^/Fe^3+^-doped enstatite crystals of com­position Mg_(2–2*x*)_Li_*x*_Fe_*x*_Si_2_O_6_ were synthesized and structurally characterized. Under the selected experimental conditions, we grew three crystals of *Pbca* orthopy­rox­ene (OPX: *x* = 0.270–0.313) and two crystals of *Pbcn* protopy­rox­ene (PPX: *x* = 0.156–0.164) using the flux-growth technique. The observed variation in the polyhedral volume and distortion of the *M*2 octa­hedron as a function of Li/Fe^3+^ doping suggests the presence of an upper limit, at least for the OPX samples. The same linear relation was observed between the polyhedral volume and 〈*M*1—O〉 bond length across all analysed samples, including the endmembers protoenstatite (PEN), orthoenstatite (OEN) and LiFe^3+^Si_2_O_6_. It seems that the *M*2 octa­hedron plays a crucial role in stabilizing the py­rox­ene topology in either the PEN or the OEN form, because the PPX and OPX samples show two distinct linear relations between the *M*2O_6_ polyhedral volume and 〈*M*2—O〉, with the PPX trend converging toward the parameters of the LiFe^3+^Si_2_O_6_ endmember, whereas the OPX trend, including OEN, diverges largely from these parameters.

## Abstract 2

Several Li^+^/Fe^3+^-doped *Pbca* orthoenstatite and *Pbcn* protoenstatite crystals are characterized revealing that varying levels of doping preferentially affect the py­rox­ene topologies.

## 1. Introduction

**Figure 1:**
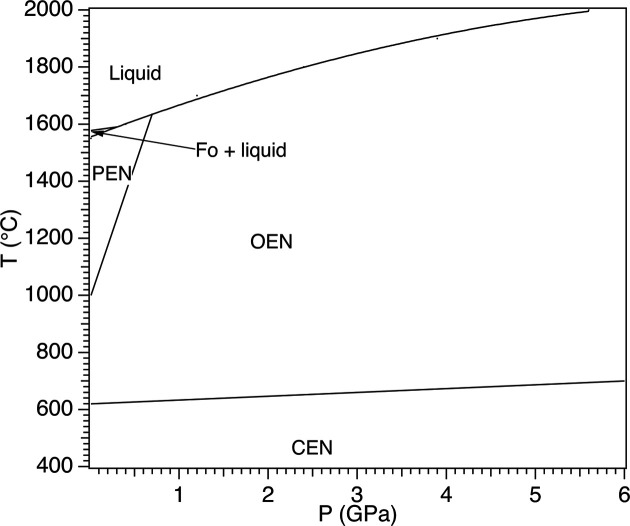
Simplified *P*–*T* diagram of Mg_2_Si_2_O_6_. Abbreviations: protoenstatite (PEN), orthoenstatite (OEN), forsterite (Fo) and clinoenstatite (CEN).

Magnesium silicate Mg_2_Si_2_O_6_ can occur in six polymorphic modifications. Two of them are ortho­rhom­bic, namely, *Pbcn* protoenstatite (PEN: Kanzaki & Xue, 2017 ▸) and *Pbca* orthoenstatite (OEN: Ganguly & Ghose, 1979 ▸; Sasaki *et al.*, 1982 ▸), whereas four monoclinic modifications have been described so far, namely, *P*2_1_/*c* low-pressure/low-tem­per­a­ture clinoenstatite (LPCEN/LTCEN: Ohashi & Finger, 1976 ▸; Ohashi, 1984 ▸; Pannhorst, 1984 ▸), *C*2/*c* (metastable) high-tem­per­a­ture clinoenstatite (HTCEN: Yoshiasa *et al.*, 2013 ▸), *C*2/*c* high-pressure clinoenstatite (HPCEN: Angel *et al.*, 1992 ▸) and *P*2_1_/*c* high-pressure clinoenstatite (HPCEN2: Lazarz *et al.*, 2019 ▸). PEN has not been found in nature and is stable in a relatively small low-pressure range (<1 GPa) at tem­per­a­tures (*T*) exceeding 1000 °C, potentially up to its incongruent melting point at 1550 °C (Boyd *et al.*, 1964 ▸). OEN has a large stability field at low pressure extending from ∼600 °C (forsterite Mg_2_SiO_4_ + liquid) up to melting (except for the small field occupied by PEN) and is the polymorphic form ubiquitously found in both igneous and metamorphic rocks. LPCEN/LTCEN is uncommon in nature, and it has been synthesized at *T* < 566 °C, clearly indicating that it is the low-tem­per­a­ture form of Mg_2_Si_2_O_6_. A simplified *P*–*T* diagram of Mg_2_Si_2_O_6_ is shown in Fig. 1 ▸. Lithium-bearing olivines (Ballirano *et al.*, 2024 ▸) were initially chosen as a test case for modelling Li^+^ + Fe^3+^ ↔ 2 Mg^2+^ coupled substitution in silicates. Next, we selected Mg_2_Si_2_O_6_ for further investigation on this issue, owing to its capability to crystallize in different space groups. We focused, in particular, on the ortho­rhom­bic polymorphs of Mg_2_Si_2_O_6_ as two synthetic *Pbcn* protopy­rox­ene crystals of Mg_(2–2*x*)_Li_*x*_Sc_*x*_Si_2_O_6_ com­position (*x* = 0.23 and 0.30) have been prepared and described so far (Smyth & Ito, 1977 ▸; Yang *et al.*, 1999 ▸), possibly suggesting that the partial coupled substitution (Li + ^VI^Me^3+^) for 2Mg plays the role of stabilizer of such a py­rox­ene topology. Despite the small differences in the corresponding ionic radii (Li^+^ = 0.760 Å and Sc^3+^ = 0.745 Å; Shannon, 1976 ▸), Li was fully ordered at the *M*2 site, whereas Sc^3+^ occupies the *M*1 site (Smyth & Ito, 1977 ▸; Yang *et al.*, 1999 ▸). However, upon recent revision of the ^VI^Li ion radius to 0.812 Å (Hawthorne & Gagné, 2024 ▸), this site preference is perfectly explainable. The latter forms a polyhedron that is smaller and much less distorted com­pared to *M*2O_6_. Owing to the significantly smaller ionic radius of Fe^3+^ com­pared to Sc^3+^ (0.649 *versus* 0.732 Å, respectively: Hawthorne & Gagné, 2024 ▸), we can hypothesize the onset of a similar ordering scheme for the Li ^+^ + Fe^3+^ ↔ 2 Mg^2+^ substitution. It is worth noting that the LiFe^3+^Si_2_O_6_ endmember com­position crystallizes as *C*2/*c* clinopy­rox­ene (Redhammer & Roth, 2004 ▸), with Li allotted at *M*2 and Fe^3+^ at *M*1. In the following, coordination polyhedra are denoted by the central cation site: thus, *M*2 octa­hedron.

According to the well-known MgO–SiO_2_ phase diagram at room pressure, the synthesis of ortho­rhom­bic enstatite requires rather high tem­per­a­tures. To decrease these tem­per­a­tures, many studies have been carried out testing different synthesis procedures, such as the sol–gel technique (Mitchell *et al.*, 1998 ▸; Ban *et al.*, 1999 ▸; Douy, 2002 ▸) or the flux method (Ito, 1975 ▸; Grandin de L’éprevier & Ito, 1983 ▸; Ushio *et al.*, 1991 ▸) to obtain enstatite and forsterite. Although the sol–gel technique opens the way to the synthesis of impurity-free films, to obtain these in a crystalline form it is still necessary to use thermal treatment. Otherwise, the flux method is preferable for obtaining larger crystals and enhance doping (Smyth & Ito, 1977 ▸). During cooling, nutrient depletion in the melt and decreased solubility can alter the original molar ratios and lead to the formation of unintended mineral phases (Bloise *et al.*, 2009 ▸; Bloise *et al.*, 2011 ▸). As reported previously, Mg_2_Si_2_O_6_ may crystallize as three polymorphs, leading to further com­plications: PEN, stable at high tem­per­a­tures (1000–1575 °C), and OEN and LTCEN stable at lower tem­per­a­tures, the extent of crystallization of which has been reported as depending on the cooling rate (Smyth, 1974 ▸; Ito, 1975 ▸; Catalano *et al.*, 2014 ▸). Consequently, slight variations in the synthesis conditions or the molar ratios can lead to the formation of additional phases, making it challenging to achieve stoichiometric control of the single phase due to the particular thermodynamic and kinetic conditions required.

For this work, crystals were grown using the flux-growth technique with lithium–vanadomolybdate as the melting agent (Ozima, 1982 ▸; Ozima & Akimoto, 1983 ▸; Grandin de L’éprevier & Ito, 1983 ▸). The acidity of the flux is crucial for enhancing the solubility of SiO_2_ by converting it into orthosilicic acid Si(OH)_4_. This can be done using MoO_3_ and V_2_O_5_ together. Fluxes lacking either vanadate or molybdate are ineffective in solubilizing SiO_2_ (Smyth & Ito, 1977 ▸; Ushio *et al.*, 1991 ▸). Indeed, MgO and SiO_2_ exhibit minimal mutual reactivity, while the presence of silicic acid promotes the formation of Si—O—Mg bonds, hence favouring the formation of phases such as enstatite and forsterite (Douy, 2002 ▸; Gu *et al.*, 2018 ▸; Bloise *et al.*, 2009 ▸). The formation of LiFeSi_2_O_6_, which begins at 500 °C when CO_2_ is released from the dissolution of Li_2_CO_3_ (Tanaka & Takei, 1997 ▸), plays a crucial role in lowering the formation tem­per­a­ture of py­rox­enes and is responsible for their doping. This effect, as reported previously (*e.g.* Ito, 1975 ▸; Grandin de L’éprevier & Ito, 1983 ▸; Smyth & Ito, 1977 ▸), stabilizes PEN (the high-tem­per­a­ture polymorph of enstatite), thereby extending its stability range to lower tem­per­a­tures.

The present work investigates the crystal chemistry of the Li^+^ + Fe^3+^ ↔ 2 Mg^2+^ coupled substitution along the Mg_2_Si_2_O_6_–LiFe^3+^Si_2_O_6_ com­positional joint by single-crystal X-ray diffraction (SCXRD). The incorporation of Li and Fe^3+^ can significantly influence the properties and behaviour of enstatite crystals, with possible implications as cathode materials for lithium-ion batteries (LiBs), given the inter­est generated by the first report on the electrochemical and structural properties of the py­rox­ene-type LiFeSi_2_O_6_ by Zhou *et al.* (2014 ▸).

## 2. Experimental

### 2.1. Synthesis

The route commonly used to synthesize Fe-doped enstatite crystals, ideally Fe_0.2_Mg_1.8_Si_2_O_6_, was followed. Granular quartz (SiO_2_; code No. 364011), magnesium oxide (MgO; code No. 459586), metallic iron (Fe; code No. 451377) and hematite (Fe_2_O_3_; code No. 451824) from Carlo Erba (reagent grade with purity ≥ 98%) were used as the starting materials without further purification.

Pre-heating was necessary to enhance the reactivity between the starting materials: granular quartz was converted into cristobalite by heating the powdered SiO_2_ to 1400 °C for 12 h, while MgO, Fe and Fe_2_O_3_ powders were heated for a week at 110 °C, to ensure com­plete dehydration. Iron(II) oxide was prepared through partial reduction of hematite by metallic iron, following the reaction: 1/3Fe + 1/3Fe_2_O_3_ = FeO. Molybdenum(VI) oxide (MoO_3_; code No. 267856), vanadium(V) oxide (V_2_O_5_; code No. 223794) and lithium carbonate (Li_2_CO_3_; code No. 62470) from Sigma–Aldrich (reagent grade with purity ≥98%) were used as flux. The flux com­position was as follows: MoO_3_ = 55.9 wt%, V_2_O_5_ = 9.8 wt% and Li_2_CO_3_ = 34.3 wt% (Bloise *et al.*, 2011 ▸). Approximately 1.25 g of finely powdered starting materials (grain size < 0.177 mm), prepared according to the ideal Fe_0.2_Mg_1.8_Si_2_O_6_ stoichiometry of Fe-doped enstatite, along with flux, were loaded into a 100 ml platinum crucible and placed in a vertical furnace. The lithium–vanadomolybdate flux was added to the starting materials/flux, maintaining a consistent starting materials (g)/ flux (g) ratio of 0.5.

Iron-doped enstatite crystals were grown in a furnace equipped with a Super Kanthal heating element (0–1700 °C), with tem­per­a­ture control provided by PtRh–PtRh thermocouples, with a precision of ±4 °C.

The thermal run proceeded as follows: a steep increment up to 1050 °C was followed by 100 h where the tem­per­a­ture was kept constant to bring about com­plete dissolution and homogenization of the mixture. The resulting melt was then cooled slowly to 650 °C at a rate of 1.25 °C h^−1^, followed by rapid quenching to room tem­per­a­ture by immersion of the crucible in water.

The growth conditions for enstatite followed well-established protocols from the literature (Bloise *et al.*, 2011 ▸; Catalano *et al.*, 2014 ▸; Catalano *et al.*, 2015 ▸). As a result, orthopy­rox­ene crystals with the com­position Mg_(2–2*x*)_Li_*x*_Fe^3+^_*x*_Si_2_O_6_ (0.15 < *x* < 0.31) were obtained. Euhedral colourless crystals, averaging 800 µm in length, were separated from the solidified flux by sonication in hot water. The crystals were recovered using a binocular microscope, selected and subsequently characterized by SCXRD.

### 2.2. Single-crystal X-ray diffraction

Five crystal fragments (labelled as **1**, **2**, **3**, **4a** and **4d**) were selected for X-ray diffraction measurements on a Bruker Kappa APEXII single-crystal diffractometer (Sapienza University of Rome, Earth Sciences Department), equipped with a CCD area detector (6.2 × 6.2 cm active detection area, 512 × 512 pixels) and a graphite-crystal monochromator, using Mo *K*α radiation from a fine-focus sealed X-ray tube. The sample-to-detector distance was 4 cm. Preliminary scrutiny of the reciprocal lattice of the samples clearly indicated their ortho­rhom­bic symmetry. Samples **1**, **2** and **3** have *Pbca* symmetry (*a* ∼ 18.17 Å, *b* ∼ 8.77 Å and *c* ∼ 5.19 Å, *i.e.* that of orthopy­rox­enes OPX) and samples **4a** and **4d** have *Pbcn* symmetry (*i.e.* that of protopy­rox­enes PPX), showing a halved *a* parameter. Diffraction data for **1**, **2**, **4a** and **4d** were collected up to sin θ_max_/λ = 1.000 Å^−1^ and those for **3** up to sin θ_max_/λ = 1.184 Å^−1^.

A total of 1708 (and 1365 for sample **3**) exposures (step = 0.4°, time/step = 15 s) covering a full reciprocal sphere with a com­pleteness > 96% and redundancy of approximately 5 were collected. Final unit-cell parameters were refined using the *SAINT* program (Bruker, 2016 ▸) with numbers of reflections ranging between 3347 and 9984, with *I* > 10σ(*I*) in the range 6 < 2θ < 91°. The associated intensities of all collected reflections were processed and corrected for Lorentz and background effects plus polarization, using *APEX2* software (Bruker, 2016 ▸). The data were corrected for absorption using a multi-scan method (*SADABS*; Bruker, 2016 ▸). The absorption correction led to a significant improvement in *wR*2_int_ (from about 0.04 to about 0.02).

### 2.3. Structure refinement

Structure refinements were carried out using *SHELXL2013* (Sheldrick, 2015 ▸) and *ShelXle* (Hübschle *et al.*, 2011 ▸). The starting coordinates were taken from Ganguly & Ghose (1979 ▸) for OPX (samples **1**, **2** and **3**) and from Smyth & Ito (1977 ▸) for PPX (samples **4a** and **4d**).

The key difference between the two ortho­rhom­bic py­rox­ene structures lies in their crystallographically distinct *T* and O sites. In *Pbcn* PPX, there is only one *T* site and three distinct O sites (O1, O2 and O3). In contrast, *Pbca* OPX has two distinct *T* sites (*T*1 and *T*2) and six distinct O sites (O1*a*, O1*b*, O2*a*, O2*b*, O3*a* and O3*b*). Both structures contain octa­hedrally coordinated cations at two distinct *M*1 and *M*2 sites.

The following parameters were refined: scale factor, extinction coefficient, atom coordinates, site-scattering values and anisotropic atomic displacement factors. In the starting stages of the refinements, Mg was used as the scatterer at the *M*1 and *M*2 sites. The observed excess of electron density at *M*1 and the deficiency in *M*2 indicated unequivocally the partition of Fe^3+^ at *M*1 and Li at *M*2. This scheme is analogous to that observed in Li/Sc PPX (Sc at *M*1 and Li at *M*2; Smyth & Ito, 1977 ▸). Subsequently, the *M*1 and *M*2 sites were modelled using Mg *versus* Fe and Mg *versus* Li scattering factors, respectively. A first set of refinements was done using neutral scattering curves for all atoms. Finally, following Hawthorne *et al.* (1995 ▸) and the results of Ballirano *et al.*(2021 ▸) for amphiboles and Ballirano *et al.* (2024 ▸) for Li/Fe^3+^-doped olivines, further refinements were done modelling the *T*1 and *T*2 sites using the Si^0^*versus* Si^4+^ scattering factors, whereas the anion sites were modelled with the O^0^*versus* O^2−^ scattering factors. The coefficients for analytical approximation to the scattering factors were from Table 6.1.1.4 of the *Inter­national Tables for Crystallography* (Volume C; Brown *et al.*, 2006 ▸), the only exception being those of O^2−^ which were taken from Hovestreydt (1983 ▸). A significant improvement of the statistical indicators was observed passing from neutral to partly ionized scattering curves.

In the final stages of the refinement, it was observed that the site occupancy factor (sof) of Fe at *M*1 and of Li at *M*2 were almost coincident (the sof of Li at *M*2 slightly exceeding that of Fe at *M*1: Δ = 0.003–0.013) and therefore they were constrained to be equal. The small discrepancy has been attributed to the presence of minor V^3+^ (ionic radius = 0.641 Å; Hawthorne & Gagné, 2024 ▸) replacing Fe^3+^, owing to its smaller scattering power (23 *versus* 26 e^−^). This result is an indirect proof that all iron occurs as Fe^3+^. The application of this constraint did not affect the various statistical indicators.

Table S1 reports space groups, unit-cell parameters, 2θ_max_ and sin θ_max_/λ of the various data collections, and relevant statistical indicators of the refinements. Table S2 lists the *M*1 and *M*2 site populations, the ion charges for O and Si, and the equivalent displacement parameters. Relevant bond distances and several parameters describing the extent of polyhedral distortion are reported in Table S3, and the results of a bond valence analysis in Table S4.

## 3. Results and discussion

Refinements substanti­ally confirmed the findings of Ballirano *et al.* (2021 ▸, 2024 ▸) regarding the use of partially ionized scattering curves of O and Si for tremolite and olivine to em­pirically com­pensate for perturbation of the electron density caused by the inter­action with other atoms (Table S2 in the supporting information). The refined ion charges for O and Si were in the range −1.492 to −1.381 and 0.377 to 0.720, respectively.

OPX samples were characterized by a coupled Li/Fe^3+^ substitution level in the 0.270 (1)–0.313 (1) sof range, whereas PPX samples were in the 0.156 (1)–0.164 (1) sof range. This finding suggests that, under the present experimental conditions, the PPX topology is favoured at smaller doping levels than in the case of Li/Sc^3+^, where crystals were obtained in the 0.23–0.30 sof range (Smyth & Ito, 1977 ▸; Yang *et al.*, 1999 ▸).

### 3.1. Unit-cell parameters

**Figure 2:**
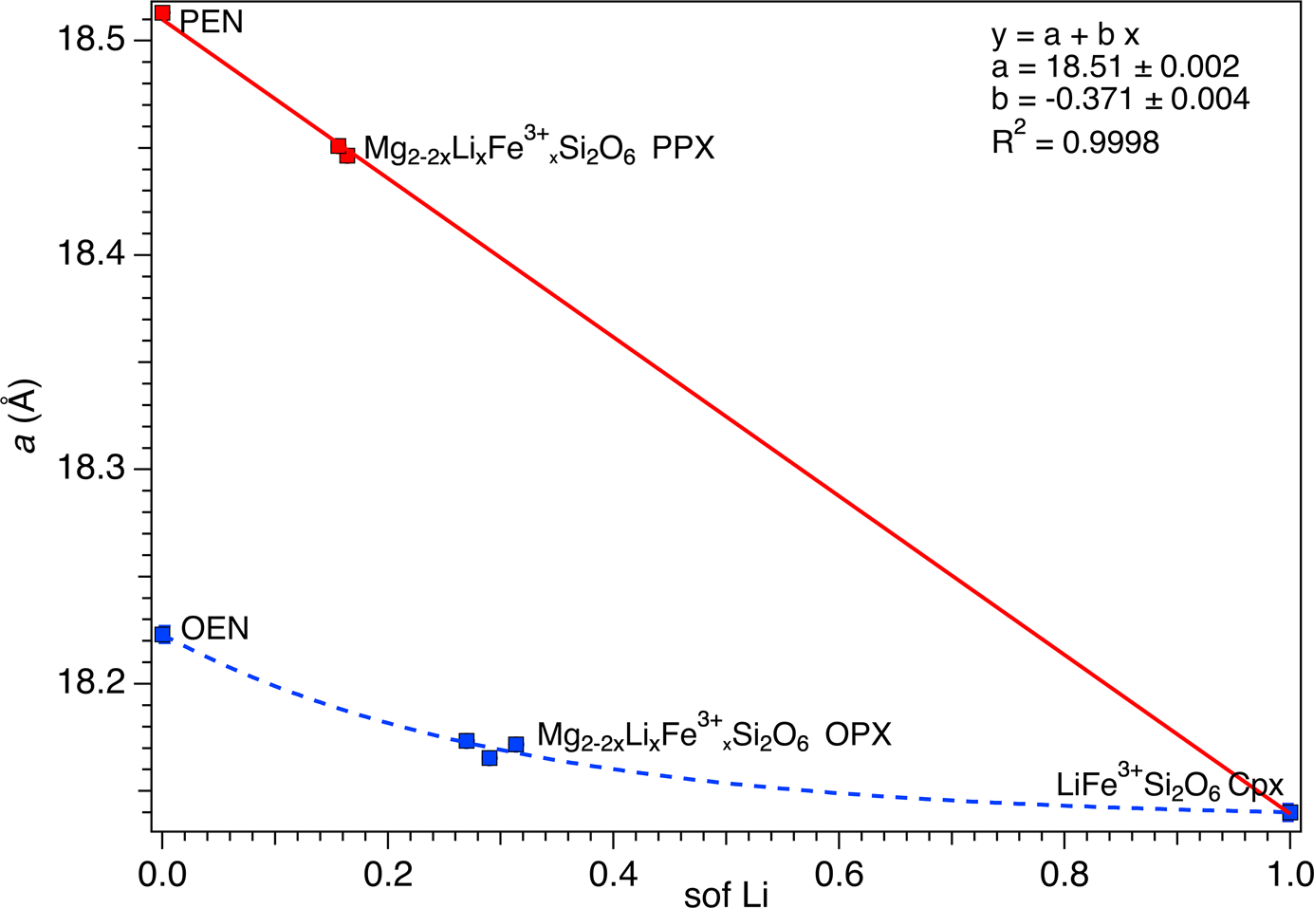
Dependence of the *a* parameter from the Li site occupancy factor (sof). The blue dashed curve is a guide to the eye showing the variation of Li in the OPX. The linear fit of the variation of Li in the PPX is reported as a red solid line. Cpx = clinopyroxene.

**Figure 3:**
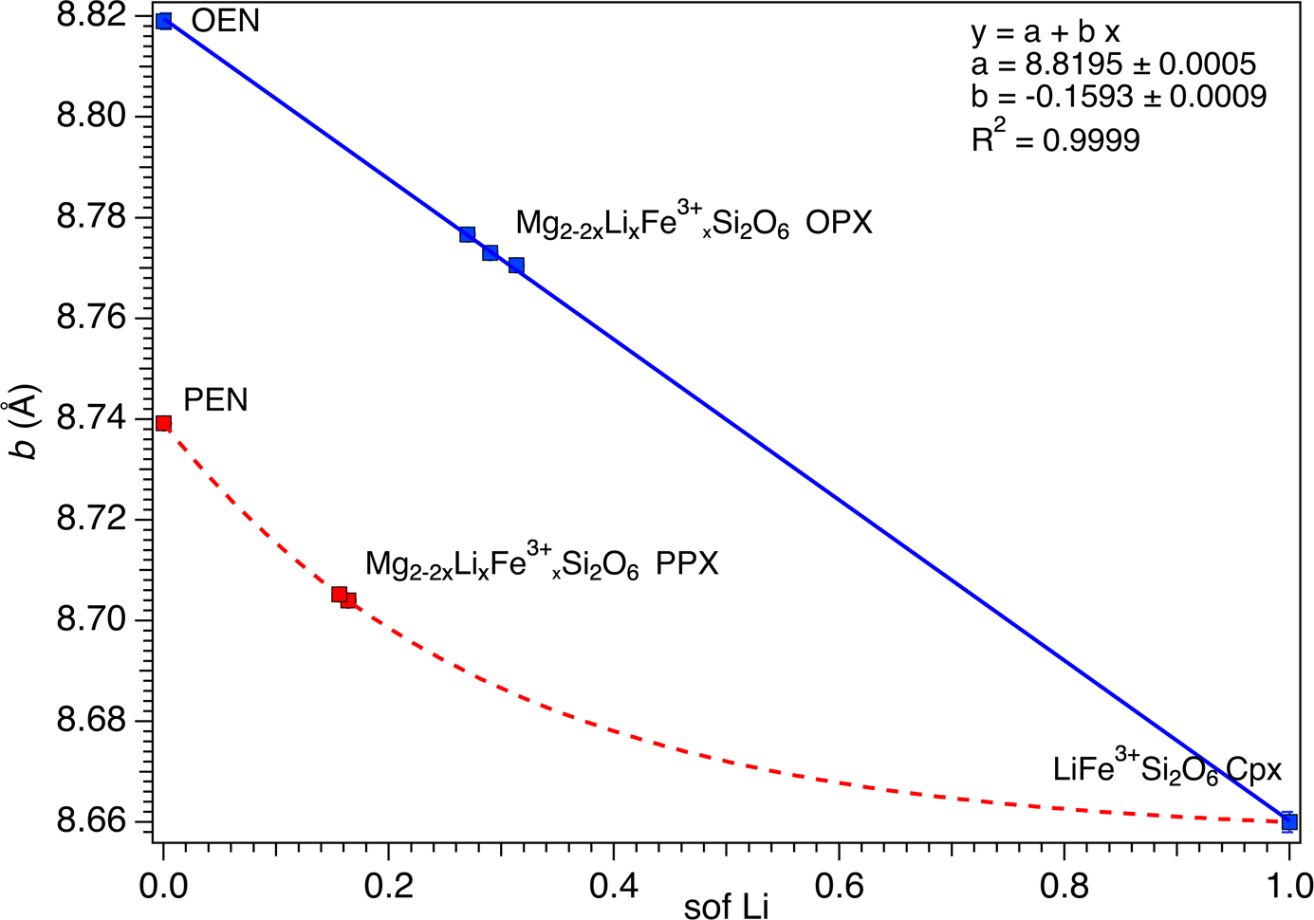
Dependence of the *b* parameter from the Li sof. The red dashed curve is a guide to the eye showing the variation of Li in the PPX. The linear fit of the variation of Li in the OPX is reported as a blue solid line.

**Figure 4:**
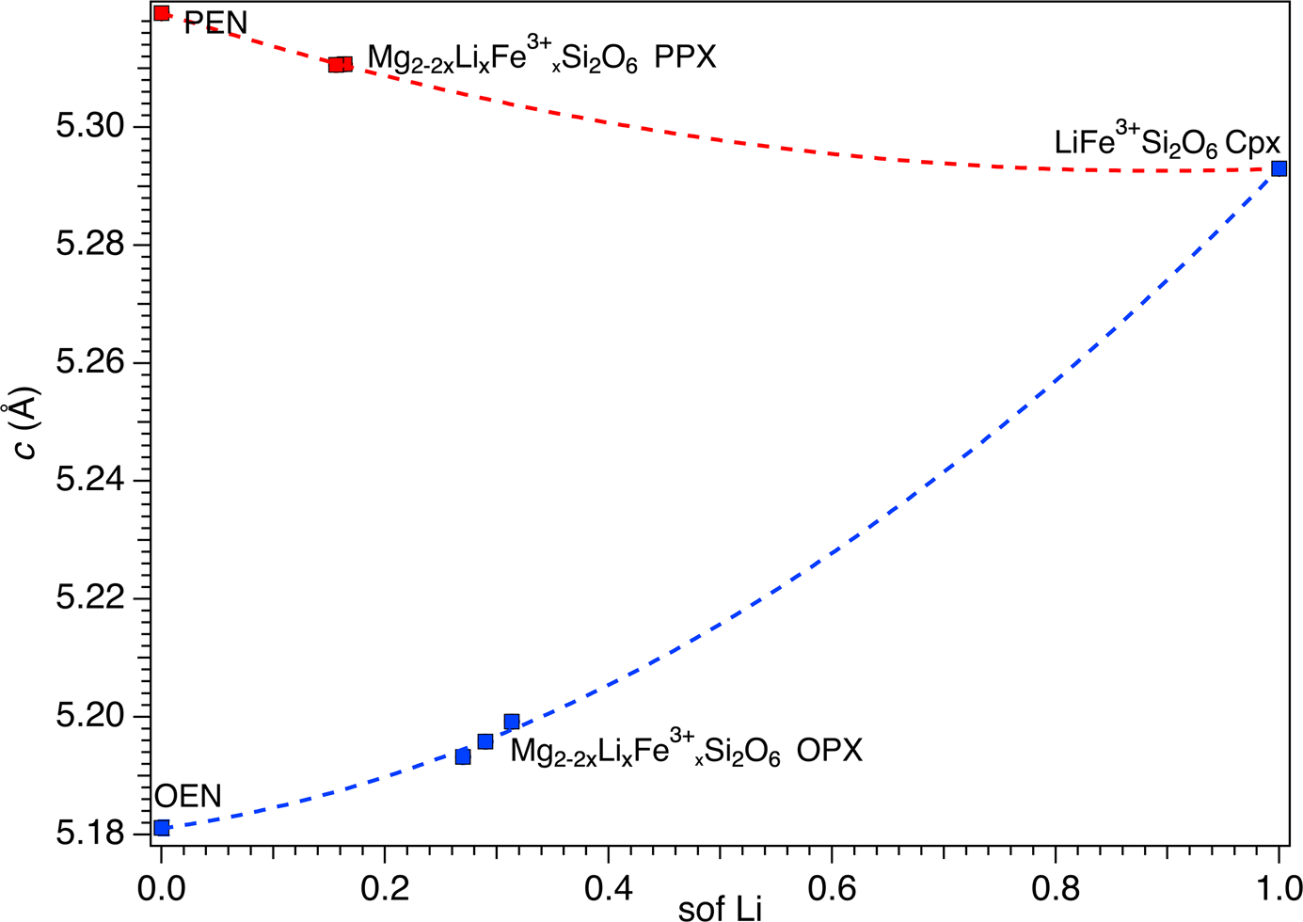
Dependence of the *c* parameter from the Li sof. The red and blue dashed curves are guides to the eye showing the variation of Li in the PPX and OPX, respectively.

Unit-cell parameters as a function of com­position are illustrated in Figs. 2 ▸ ▸–4 ▸ (see also Fig. S1 in the supporting information), where LiFe^3+^Si_2_O_6_ was considered the common endmember for both the OPX and the PPX series. To obtain a com­parable data set, the *a* parameter of LiFe^3+^Si_2_O_6_ was recalculated based on an ortho­rhom­bic cell, according to the well-known relationship *a*_orth_ = 2*a*_mon_sinβ, whereas for the PPX samples, the *a* parameter was multiplied by two, in both cases resulting in a doubled unit-cell volume. The OPX and PPX samples show a different behaviour for each unit-cell parameter, coherent with the significantly different volumes of the OEN and PEN endmembers. For OPX samples, the unit-cell volume dependence on com­position follows a bell-shaped curve (*i.e.* inter­mediate com­positions have a volume smaller than both endmembers), whereas there is a marked decrease from PEN to LiFe^3+^Si_2_O_6_ (Fig. S1). For both series, the *a* unit-cell parameters contract from Mg_2_Si_2_O_6_ to LiFe^3+^Si_2_O_6_. However, the contraction is remarkably higher and linear for the PPX samples, whereas the trend is nonlinear and decreases at a significantly smaller rate for the OPX samples (Fig. 2 ▸). The trend in reversed in the case of the *b* unit-cell parameter (Fig. 3 ▸). Conversely, the *c* unit-cell parameter shows a strong expansion from OEN to LiFe^3+^Si_2_O_6_, whereas the PPX series is characterized by a small contraction (Fig. 4 ▸). Both trends are nonlinear.

### 3.2. Structural features

Before discussing how the structural features of the two series of orthopy­rox­enes depend on their com­position, it is worth noting that reference structural data for PEN were obtained through Rietveld refinement of laboratory powder X-ray diffraction data collected in reflection mode (Kanzaki & Xue, 2017 ▸). However, no details of the refinement procedure were reported in the related article, particularly regarding the use of soft constraints on bond distances and how preferred orientation was accounted for. As a consequence, the accuracy of the structural parameters for PEN might be somewhat lower than that of the results. Therefore, any correlations drawn for PPX samples should be approached with some caution.

**Figure 5:**
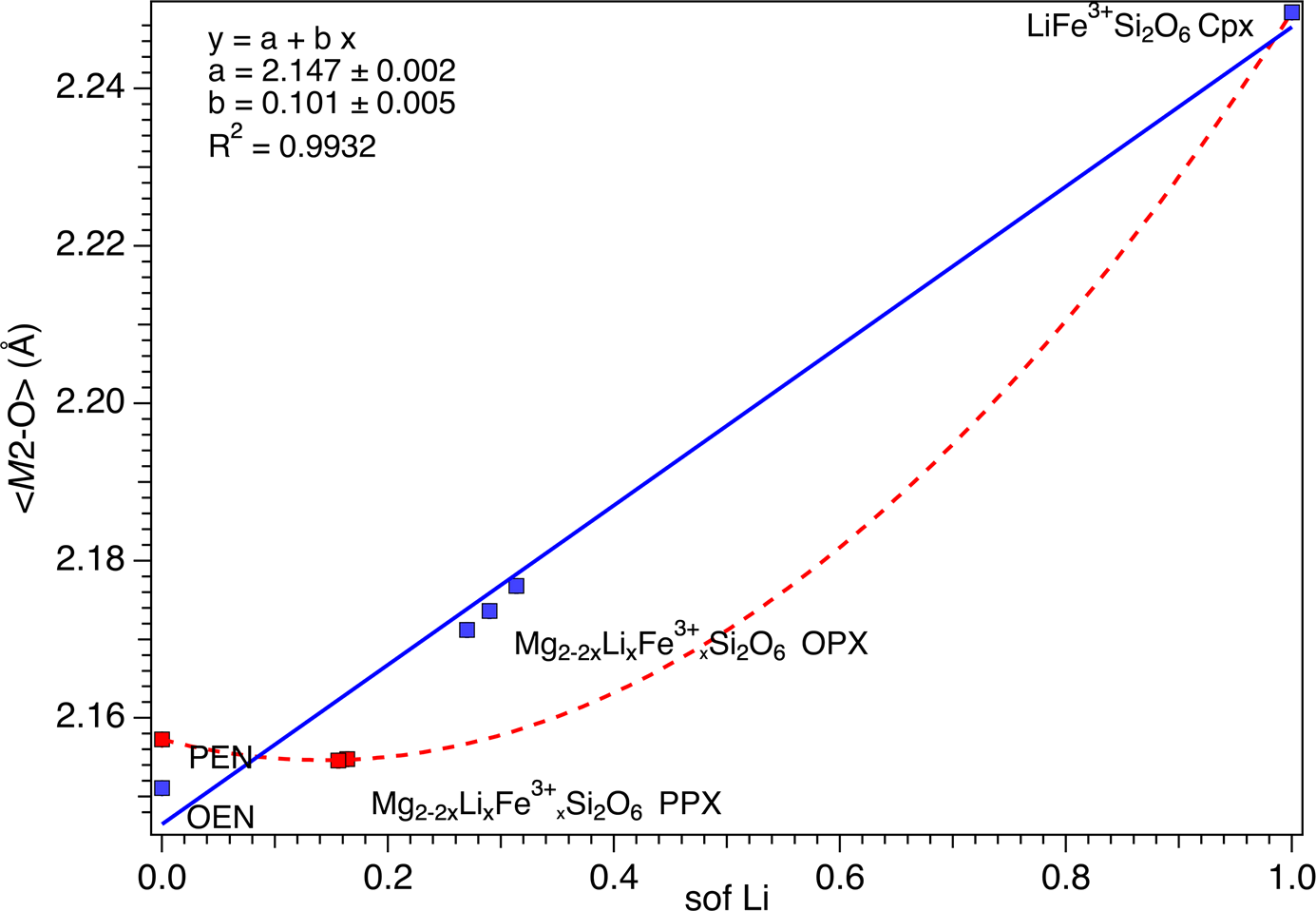
Variation of 〈*M*2—O〉 as a function of the Li sof. The red dashed curve is a guide to the eye showing the variation of Li in the PPX. The linear fit of the variation of Li in the OPX is reported as a blue solid line.

That said, unit-cell parameters and 〈*M*1—O〉 and 〈*M*2—O〉 show similar correlations with the Li/Fe^3+^ sof (Figs. 5 ▸ and 6 ▸). For the OPX samples, there is a noticeable increase of 〈*M*2—O〉 from OEM (2.151 Å) to LiFe^3+^Si_2_O_6_ (2.249 Å). In contrast, in the case of the PPX samples, PEN has a slightly larger 〈*M*2—O〉 (2.157 Å) than OEN and the doped samples do not show appreciable variations from that value (2.155 Å), but they are still significantly lower than the LiFe^3+^Si_2_O_6_ value. The 〈*M*1—O〉 decreases smoothly in both series of samples from 2.078 (OEN) or 2.089 (PEN) to 2.025 Å for LiFe^3+^Si_2_O_6_. The trend for OPX is linear and almost linear for PPX if the value for PEN is accepted as accurate.

**Figure 7:**
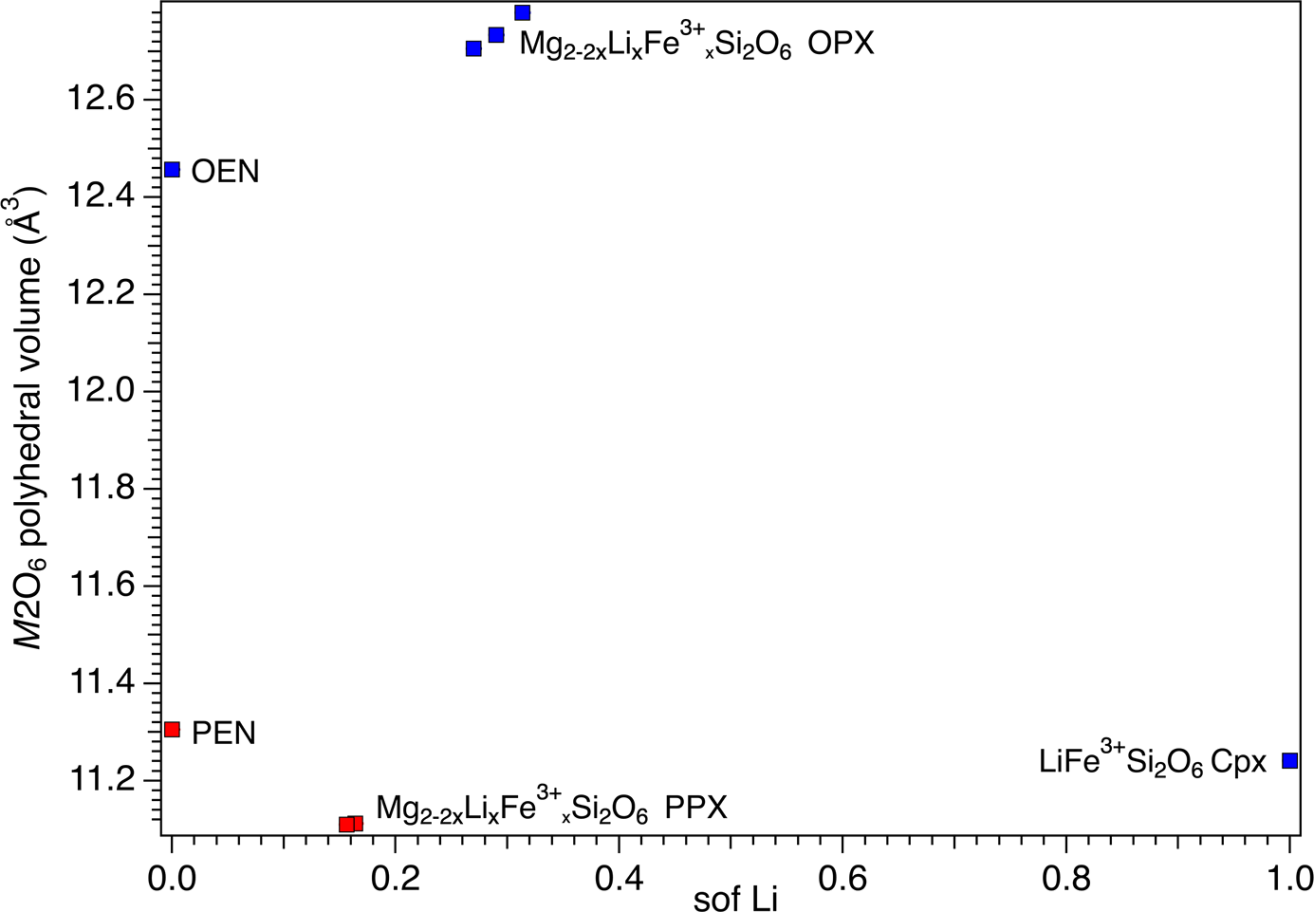
Variation of the volume of the *M*2 octa­hedron as a function of the Li sof.

**Figure 8:**
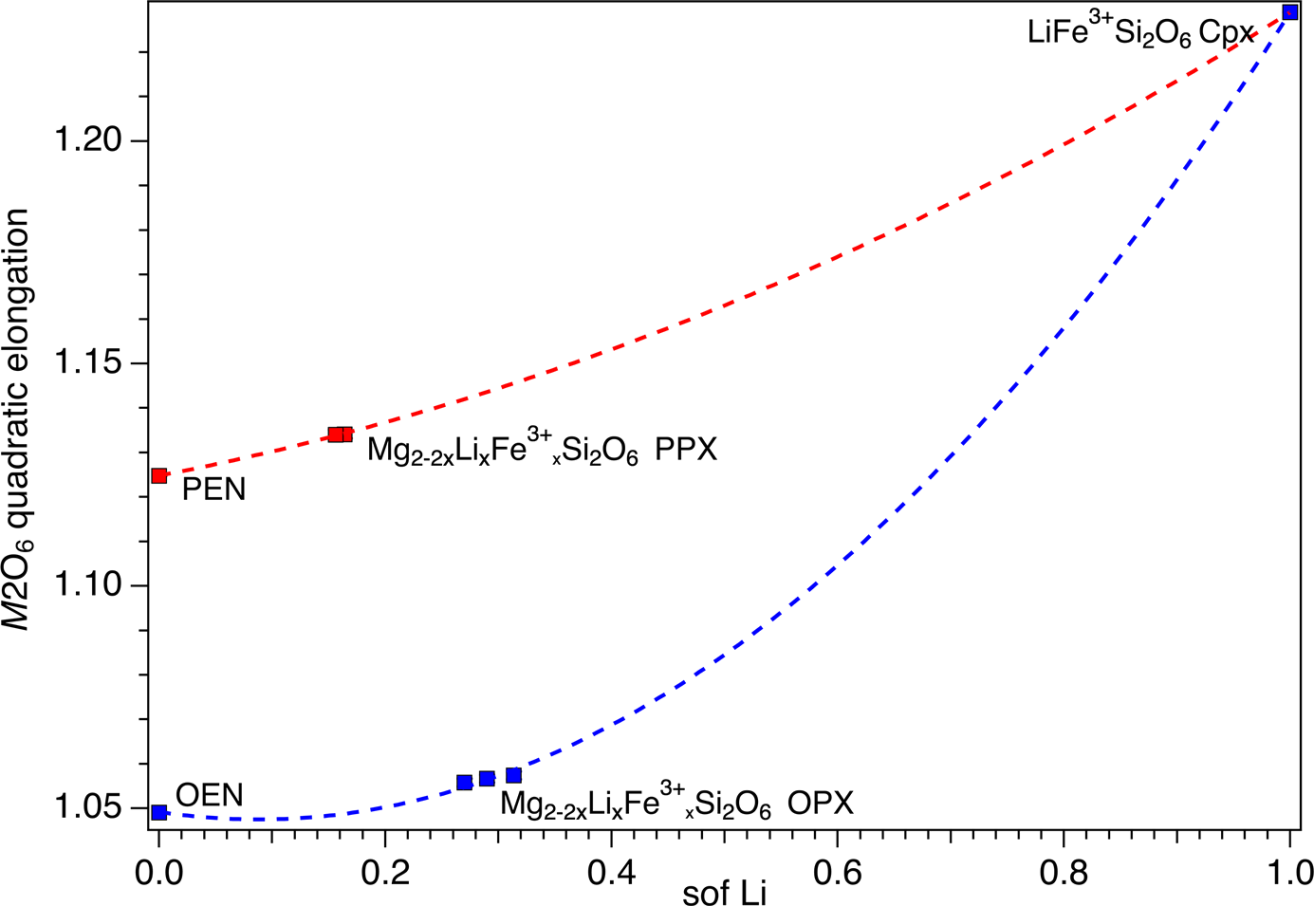
Variation of the quadratic elongation (QE) of the *M*2 octa­hedron as a function of the Li sof. The red and blue dashed curves are guides to the eye showing the variation of Li in the PPX and OPX, respectively.

**Figure 11:**
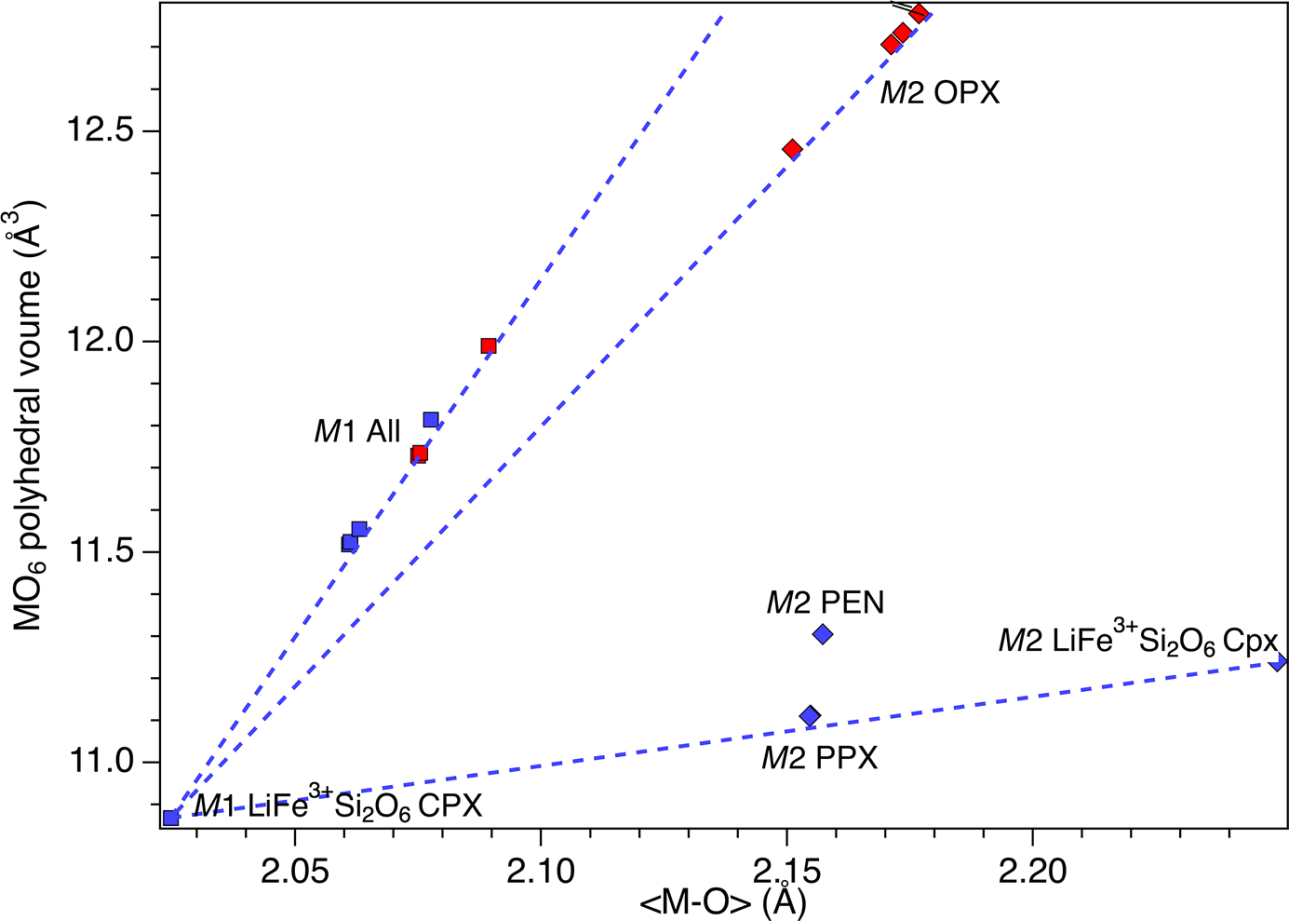
Variation of the polyhedral volume of the *M*2 and *M*1 octa­hedra as a function of 〈*M*—O〉. The dashed lines are guides to the eye. Key: red diamonds represent the *M*2O_6_ polyhedral volume for OPX; blue diamonds the *M*2O_6_ polyhedral volume for PPX, PEN and LiFe^3+^Si_2_O_6_Cpx; red squares the *M*1O_6_ polyhedral volume for OPX; blue squares the *M*1O_6_ polyhedral volume for PPX, PEN and LiFe^3+^Si_2_O_6_. Cpx = clinopyroxene.

Analysis of the volume and deviation from ideal shape of the *M*2 and *M*1 octa­hedra (Figs. 7 ▸ ▸ ▸ ▸–11 ▸) took into consideration several parameters (Table S3): polyhedral volume (Swanson & Peterson, 1980 ▸), polyhedral volume distortion (Makovicky & Balić-Žunić, 1998 ▸), distortion index (Baur, 1974 ▸), mean quadratic elongation and bond angle variance (Robinson *et al.*, 1971 ▸), and effective coordination number (Hoppe, 1979 ▸). The volume of the *M*2 octa­hedron is larger than that of the *M*1 octa­hedron for the OPX series of samples (∼12.7–12.8 Å^3^*versus* ∼11.5 Å^3^; Figs. 7 ▸ and 9 ▸). Moreover, the *M*2 octa­hedron is significantly more distorted than the *M*1 octa­hedron, as indicated by the mean quadratic elongation of ∼1.06 *versus* 1.01. This same behaviour holds true for the LiFe^3+^Si_2_O_6_ monoclinic endmember, in which the *M*2 octa­hedron has a very large quadratic elongation (1.229) typical of the Li-bearing clinopy­rox­enes (Cameron & Papike, 1981 ▸). In contrast, for the PPX samples, the polyhedral volume of the *M*2 octa­hedron is smaller than that of the *M*1 octa­hedron (∼11.1 Å^3^*versus* ∼11.7 Å^3^) and has a larger distortion, even larger than that of the OPX samples (mean quadratic elongation ∼1.13 *versus* 1.01). The smaller dimension of the *M*2 octa­hedron in the PEN and PPX samples with respect to the OPX samples is caused by the sharing of two edges of the octa­hedron with tetra­hedra for the former, whereas in the case of the OEN and OPX samples, no polyhedral edges are shared (Cameron & Papike, 1981 ▸). Comparison of Figs. 7 ▸ and 8 ▸ suggests that for OEN and OPX samples, the increase of the volume of the *M*2 octa­hedron as a function of a growing level of doping is coupled to an increase in the mean quadratic elongation. In contrast, PEN and PPX samples do not show a well-defined dependence. OPX samples show an increase of the volume of the *M*2 octa­hedron with an increased level of doping and much larger than that of the LiFe^3+^Si_2_O_6_ monoclinic endmember, whose volume is com­parable to that of PPX but with a larger mean quadratic elongation value, much larger than for the OPX and PPX samples. This difference between OPX and LiFe^3+^Si_2_O_6_ could potentially impose an upper limit on the level of coupled Li/Fe^3+^ substitution. Inter­estingly, recalculation of the original structural data of Smyth & Ito (1977 ▸) and Yang *et al.* (1999 ▸) for Li/Sc^3+^-doped PPX samples (Table S3) results in mean quadratic elongations of the *M*2 octa­hedron (where a similar Li *versus* Mg substitution occurs) that aligns almost perfectly with the curve in Fig. 8 ▸ for the present Li/Fe^3+^-doped PPX samples. In contrast, the polyhedral volume is slightly greater than that of PEN, possibly suggesting that the value reported by Kanzaki & Xue (2017 ▸) is too large. Fig. 11 ▸ shows the variation in polyhedral volume of the *M*2 and *M*1 octa­hedra in Li/Fe^3+^-doped orthopy­rox­enes as a function of 〈*M*—O〉. As can be seen, the volume of the *M*1 octa­hedron is linearly correlated with 〈*M*—O〉 for both OPX and PPX samples. Conversely, two separate dependences, one for OPX and one for PPX samples, are observed for the *M*2 octa­hedron. The displaced position of PEN from this trend suggests that the polyhedral volume of PEN is wrong (too large) and may possibly be related to a lower accuracy of the bond distances as arising from powder X-ray diffraction data with respect to the rest of the data set which is derived from single-crystal SREF.

Fig. S2 and Table S4 report the dependence of the bond valence sum at the various O sites. The parameters used for the calculations were taken from Gagné & Hawthorne (2015 ▸). Trends are clearly seen for the analysed samples and there are positive and negative deviations from the valence-sum rule.

## 4. Conclusions

In this study, we have investigated the crystal structure and doping of enstatite crystals with Li and Fe^3+^. The incorporation of these dopants can significantly influence the properties and behaviour of enstatite crystals, making them inter­esting. Under the experimental conditions, we recovered five samples of Mg_(2–2*x*)_Li_*x*_Fe^3+^_*x*_Si_2_O_6_ py­rox­enes, namely, three ortho­pyrox­ene (OPX) with 0.270 < *x* < 0.313 and two clinopy­rox­ene (PPX) with 0.156 < *x* < 0.164. This shows that varying levels of doping preferentially affect the py­rox­ene topologies.

Analysis of the ship between the volume and distortion of the *M*2 octa­hedron *versus* the level of doping indicates a possible upper limit for the coupled substitution of (Li + Fe^3+^) for Mg, at least for OPX. The significant role of this polyhedron in stabilizing a specific py­rox­ene topology is further supported by the observation that for all analysed samples (including the endmembers PEN, OEN and LiFe^3+^Si_2_O_6_), the relationship between the polyhedral volume and 〈*M*1—O〉 is linear. Conversely, there are two distinct linear relations for PPX and OPX samples between the *M*2O_6_ polyhedral volume and 〈*M*2—O〉, with only the PPX trend converging toward the LiFe^3+^Si_2_O_6_ endmember. Notably, the smaller *M*2 octa­hedron in PPX shares two edges with Si tetra­hedra, whereas the larger *M*2 octa­hedron in OPX does not share two edges with Si tetra­hedra.

This research enhances the understanding of crystal structure and doping in enstatite crystals, suggesting a potential use of Li/Fe^3+^-doped enstatite in energy storage devices where a very stable structural framework is required for long-term Li^+^ ion extraction/insertion.

## 5. Related literature

The following references are cited in the supporting information: Ilinca (2022 ▸); Momma & Izumi (2011 ▸).

## Supplementary Material

Crystal structure: contains datablock(s) OPX_1, OPX_2, OPX_3, PPX_4a, PPX_4d. DOI: 10.1107/S2052520624011624/ra5147sup1.cif

Structure factors: contains datablock(s) OPX_1. DOI: 10.1107/S2052520624011624/ra5147OPX_1sup2.hkl

Structure factors: contains datablock(s) OPX_2. DOI: 10.1107/S2052520624011624/ra5147OPX_2sup3.hkl

Structure factors: contains datablock(s) OPX_3. DOI: 10.1107/S2052520624011624/ra5147OPX_3sup4.hkl

Structure factors: contains datablock(s) PPX_4a. DOI: 10.1107/S2052520624011624/ra5147PPX_4asup5.hkl

Structure factors: contains datablock(s) PPX_4d. DOI: 10.1107/S2052520624011624/ra5147PPX_4dsup6.hkl

Supporting information file. DOI: 10.1107/S2052520624011624/ra5147sup7.pdf

CCDC references: 2406244, 2406245, 2406246, 2406247, 2406248

## Figures and Tables

**Figure 6:**
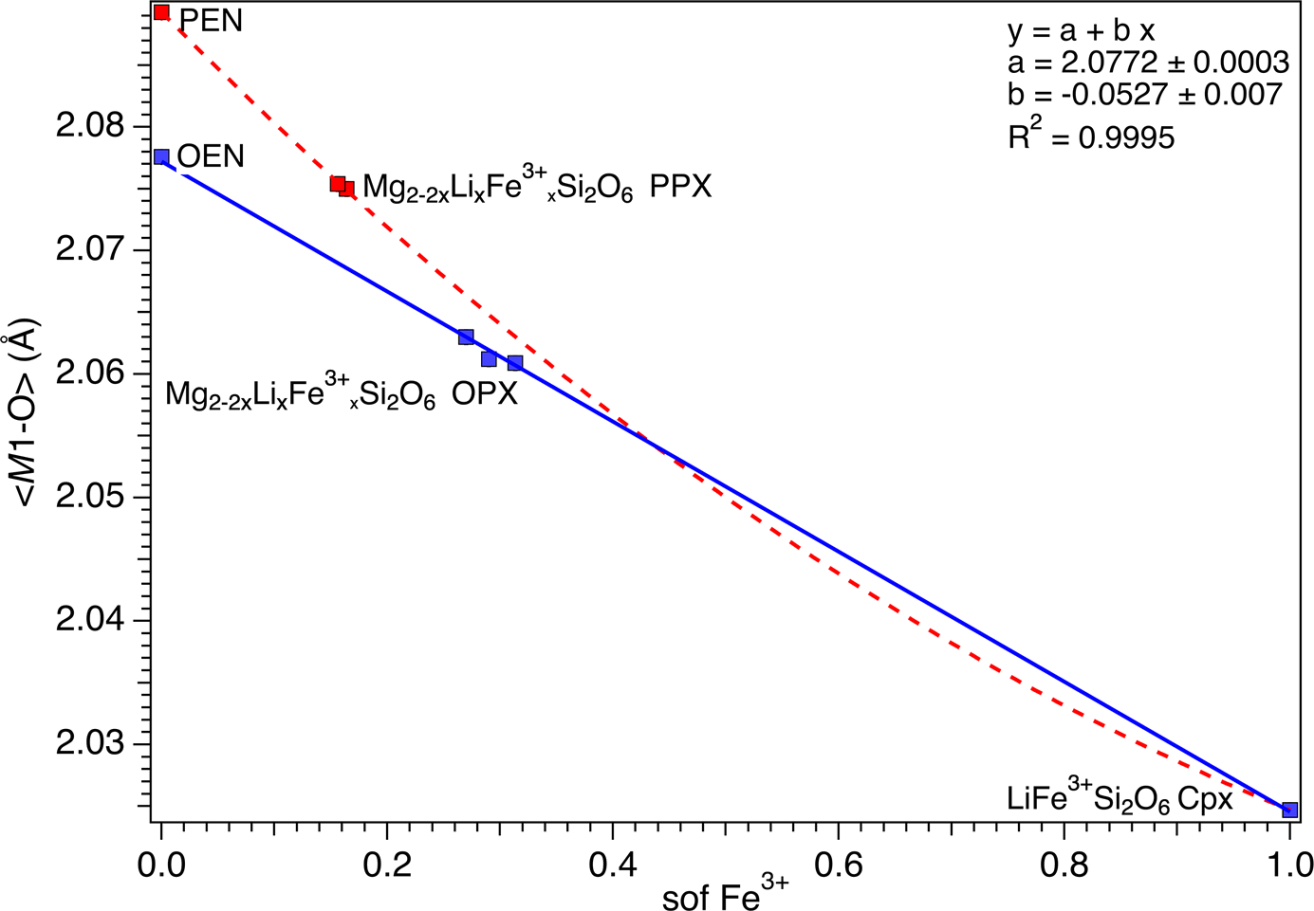
Variation of 〈*M*1—O〉 as a function of the Fe^3+^ sof. The red dashed curve is a guide to the eye showing the variation of Fe^3+^ in the PPX. The linear fit of the variation of Fe^3+^ in the OPX is reported as a blue solid line.

**Figure 9:**
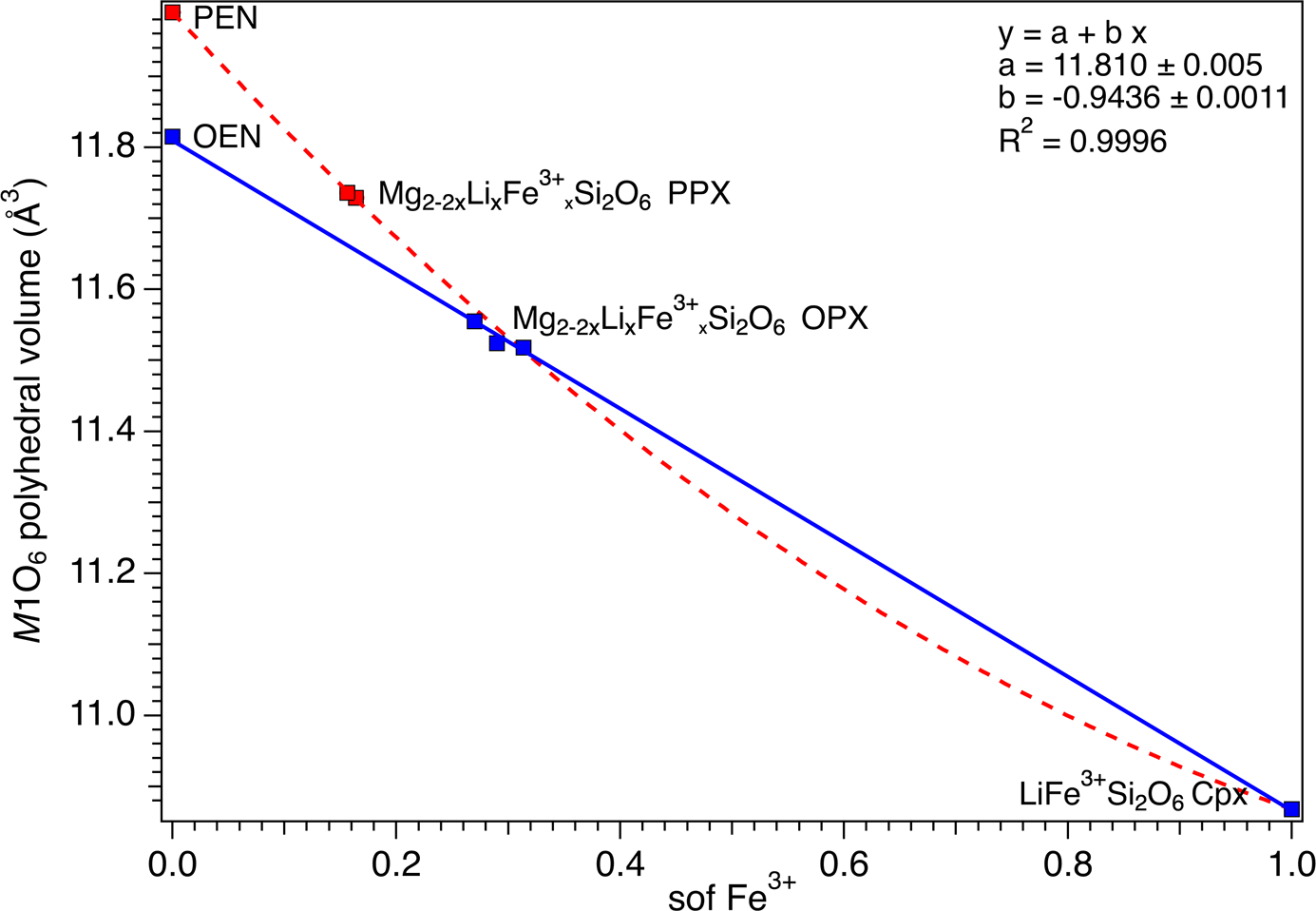
Variation of the volume of the *M*1 octa­hedron as a function of the Fe^3+^ sof. The red dashed curve is a guide to the eye showing the variation of Fe^3+^ in the PPX. The linear fit of the variation of Fe^3+^ in the OPX is reported as a blue solid line.

**Figure 10:**
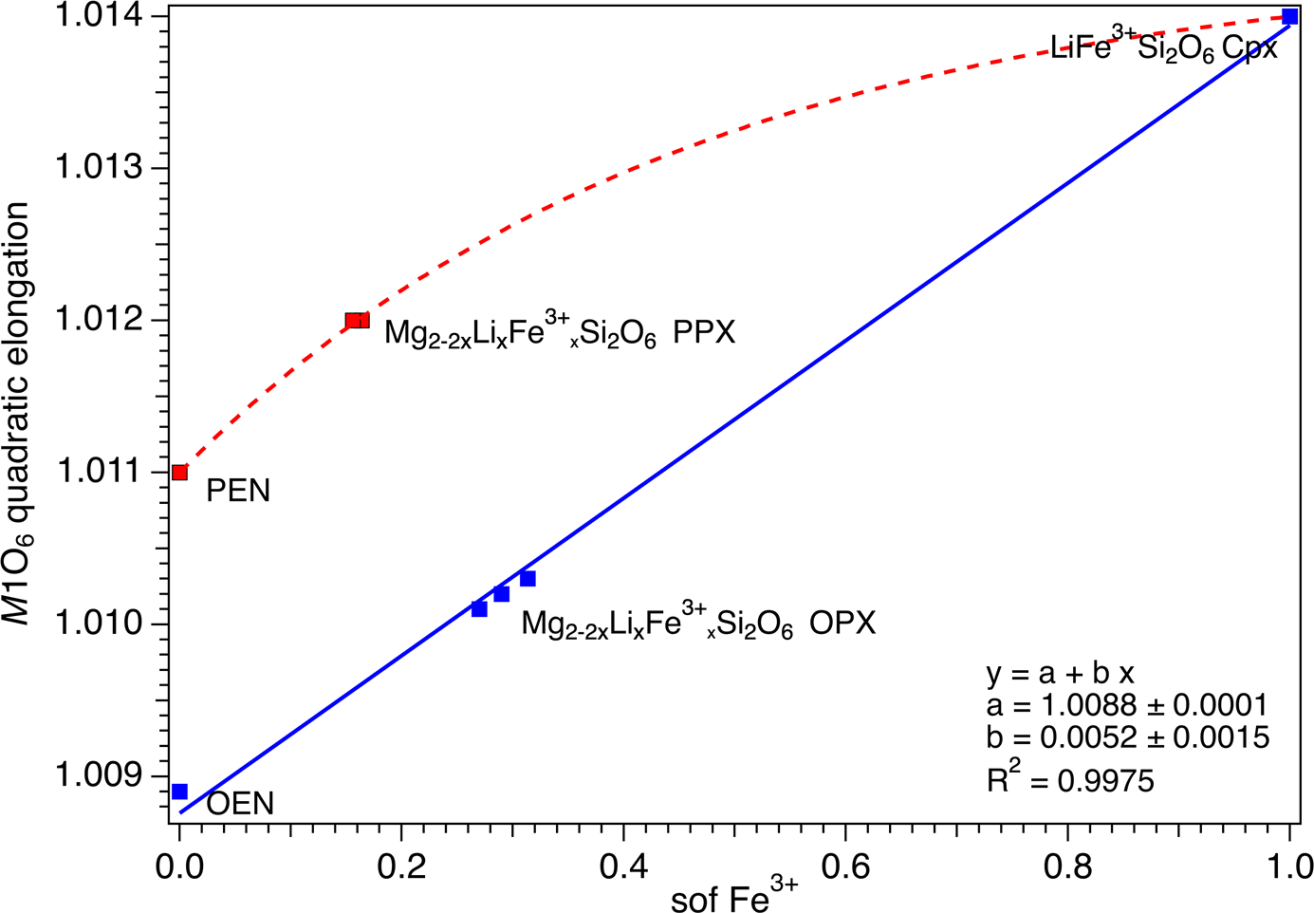
Variation of the quadratic elongation (QE) of the *M*1 octa­hedron as a function of the Li sof. The red dashed curve is a guide to the eye showing the variation of Fe^3+^ in the PPX. The linear fit of the variation of Fe^3+^ in the OPX is reported as a blue solid line.
