# Supplementary material for: Crystal structure and doping in synthetic enstatite: an analysis of Li/Fe3+-doped single-crystal samples
Source: Acta Crystallogr B Struct Sci Cryst Eng Mater. 2025 Jan 14;81(Pt 1):84–91. doi: 10.1107/S2052520624011624 (PMC11801706; doi:10.1107/S2052520624011624)

Acta Cryst B

STRUCTURAL SCIENCE
CRYSTAL ENGINEERING
MATERIALS

Volume 81 (2025)

Supporting information for article:

Crystal structure and doping in synthetic enstatite: an analysis of Li/Fe^{3+}-doped single-crystal samples

Paolo Ballirano, Beatrice Celata, Alessandro Pacella, Andrea Bloise and Ferdinando Bosi

Supporting information

Table S1 Miscellaneous data of the refinements of Li-Fe^{3+}-doped ortho (1, 2, 3) and protopyroxenes (4a and 4d). Data of orthoenstatite, protoenstatite, Li-Sc-protopyroxene, and $LiFeSi_2O_6$ from literature are reported for comparison

	orthoenstatite§	1	2	3	4a	4d	protoenstatite†	Li-Sc-protopx‡	$LiFeSi_2O_6$#
Space group	*Pbca*	*Pbca*	*Pbca*	*Pbca*	*Pbcn*	*Pbcn*	*Pbcn*	*Pbcn*	*C2/c*
a (Å)	18.223(4)	18.1735(4)	18.1654(4)	18.1718(4)	9.2232(2)	9.2255(2)	9.25655(9)	9.2557(5)	9.664(2)
$2a_{mon}$ sin β (Å)	-	-	-	-	-	-	-	-	18.140(4)
b (Å)	8.8190(16)	8.77666(18)	8.7730(2)	8.7706(2)	8.7040(2)	8.7052(2)	8.73919(9)	8.7648(7)	8.660(2)
c (Å)	5.1812(13)	5.19316(10)	5.19580(10)	5.19920(10)	5.3107(10)	5.3106(10)	5.31939(5)	5.3331(2)	5.293(1)
β (°)	-	-	-	-	-	-	-	-	110.19(3)
V ($Å^3$)	832.66(10)	828.32(4)	828.03(5)	828.64(5)	426.34(2)	426.49(2)	430.311(5)	432.64(4)	415.78(5)/ 831.56(10)**
$2\theta_{max}$ (°)	75	114.55	90.59	90.59	90.59	90.64	***	60	55.84
$\sin\theta/\lambda_{max}$ ($Å^{-1}$)	0.857	1.184	1.000	1.000	1.000	1.000	***	0.704	0.659
R_{int}	-	0.0188	0.0283	0.0196	0.0153	0.0236	***	0.0090	0.0284
wR_2 all	0.0310*	0.0500	0.0420	0.0406	0.0354	0.0399	***	0.0180	0.0568
*R*1 > for $I > 2\sigma$ (n. ref.)	-	0.0280 (4934)	0.0207 (2907)	0.0188 (3231)	0.0141 (1661)	0.0181 (1539)	***	-	0.0149 (-)
*R*1 all (n. ref.)	0.0210 (2214)	0.0324 (5429)	0.0296 (3483)	0.0212 (3458)	0.0155 (1753)	0.0232 (1760)	***	0.0170(262)	0.0175 (3173)

§ = Ganguly & Ghose (1979)

[†] Kanzaki & Xue (2017)

[‡] Yang et al. (1999)

[‡‡] Smyth & Ito (1977)

[#] Redhammer & Roth (2004)

[*] *wR*.

[**] Volume of the corresponding orthorhombic cell.

[***] Rietveld refinement.

Table S2 Site occupancies at *M*1 and *M*2, ion charge for O and Si (Ballirano et al., 2021), and U_{eq} of Li-Fe^{3+}-doped ortho (1, 2, 3) and protopyroxenes (4a and 4d). Data of orthoenstatite, protoenstatite, Li-Sc-protopyroxene and $LiFeSi_2O_6$ from literature are reported for comparison

	orthoenstatite[§]	1	2	3	4a	4d	protoenstatite[†]	Li-Sc-protopx[‡]	$LiFeSi_2O_6$[#]
*M*1 occupancy	Mg	$Mg_{0.730(1)}Fe^{3+}_{0.270}$	$Mg_{0.710(1)}Fe^{3+}_{0.290}$	$Mg_{0.687(1)}Fe^{3+}_{0.313}$	$Mg_{0.836(1)}Fe^{3+}_{0.164}$	$Mg_{0.844(1)}Fe^{3+}_{0.156}$	Mg	$Mg_{0.77(0)}Sc^{3+}_{0.23}$	Fe^{3+}
*M*2 occupancy	Mg	$Mg_{0.730(1)}Li_{0.270}$	$Mg_{0.710(1)}Li_{0.290}$	$Mg_{0.687(1)}Li_{0.313}$	$Mg_{0.836(0)}Li_{0.164}$	$Mg_{0.844(0)}Li_{0.156}$	Mg	$Mg_{0.77(0)}Li_{0.23}$	Li
Ion charge Si	-	0.720	0.517	0.457	0.392	0.377	-	-	-
Ion charge O	-	-1.407	-1.381	-1.492	-1.427	-1.403	-	-	-
*M*1 U_{eq} (Å^2)	0.00550(5)	0.00574(3)	0.00580(3)	0.00581(3)	0.00580(4)	0.00594(5)	0.0163(8)	0.0068(4)	0.0035(1)
*M*2 U_{eq} (Å^2)	0.00738(6)	0.00774(5)	0.00829(6)	0.00829(5)	0.00691(5)	0.00705(6)	0.0163	0.0092(6)	0.0143(8)
*T*1 U_{eq} (Å^2)	0.00422(4)	0.00374(2)	0.00398(3)	0.00393(3)	0.00417(3)	0.00435(3)	0.0071(6)	0.0062(4)	0.0033(1)
*T*2 U_{eq} (Å^2)	0.00422(4)	0.00384(2)	0.00408(3)	0.00404(3)					
O1a U_{eq} (Å^2)	0.00571(10)	0.00568(4)	0.00596(6)	0.00587(5)	0.00573(4)	0.00585(5)	0.0120(8)	0.0063(6)	0.0046(2)
O1b U_{eq} (Å^2)	0.00687(10)	0.00577(4)	0.00581(6)	0.00592(5)					
O2a U_{eq} (Å^2)	0.00589(9)	0.00675(5)	0.00691(6)	0.00688(5)	0.00805(4)	0.00816(6)	0.0120	0.0100(8)	0.0081(2)
O2b U_{eq} (Å^2)	0.00668(10)	0.00742(5)	0.00763(6)	0.00767(5)					
O3a U_{eq} (Å^2)	0.00677(10)	0.00673(5)	0.00689(6)	0.00696(6)	0.00821(4)	0.00827(6)	0.0120	0.0076(8)	0.0084(2)
O3b U_{eq} (Å^2)	0.00639(10)	0.00706(5)	0.00733(6)	0.00744(5)					

[§] = Ganguly & Ghose (1979)
[†] Kanzaki & Xue (2017)
[‡] Yang et al. (1999)
[‡‡] Smyth & Ito (1977)
[#] Redhammer & Roth (2004)

Table S3 Selected bond distances (Å) of the analysed crystals of Li-Fe^{3+}-doped ortho (1, 2, 3) and protopyroxenes (4a and 4d). Data of orthoenstatite, protoenstatite, Li-Sc-protopyroxene and $LiFeSi_2O_6$ from literature are shown for comparison

	orthoenstatite[§]	1	2	3
*M*1-O2a	2.0059(6)	1.9658(4)	1.9633(4)	1.9613(4)
*M*1-O2b	2.0462(6)	2.0059(4)	2.0020(4)	2.0000(4)
*M*1-O1a	2.0276(7)	2.0273(4)	2.0262(4)	2.0276(4)
*M*1-O1b	2.0656(7)	2.0461(4)	2.0450(4)	2.0447(4)
*M*1-O1a	2.1497(6)	2.1514(4)	2.1508(4)	2.1512(4)
*M*1-O1b	2.1707(6)	2.1812(4)	2.1800(4)	2.1803(4)
<*M*1-O>	2.0776	2.0630	2.0612	2.0609
<i.r.> (Å)[d]	0.723	0.703	0.702	0.700
Poly. Vol.[e]	11.815	11.555	11.524	11.518
Poly. Vol. Dist.[f]	0.011	0.009	0.009	0.009
DI[g]	0.0265	0.0334	0.0337	0.0339
QE[h]	1.0089	1.0101	1.0102	1.0103
σ_θ^{2} [i]	26.8351	29.41	29.75	29.91
ECoN[j]	5.830	5.725	5.719	5.714
*M*2-O2b	1.9925(7)	2.0136(5)	2.0157(5)	2.0191(4)
*M*2-O2a	2.0319(7)	2.0450(5)	2.0477(5)	2.0500(4)
*M*2-O1b	2.0555(6)	2.0605(5)	2.0607(5)	2.0623(4)
*M*2-O1a	2.0884(6)	2.1020(5)	2.1033(5)	2.1046(4)
*M*2-O3a	2.2904(7)	2.2934(5)	2.2938(5)	2.2949(4)

*M*2-O3b	2.4478(7)	2.5125(5)	2.5203(5)	2.5298(5)
<*M*2-O>	2.1511	2.1712	2.1736	2.1768
<i.r.> (Å)[d]	0.723	0.747	0.749	0.751
Poly. Vol.[e]	12.457	12.706	12.734	12.780
Poly. Vol. Dist.[f]	0.065	0.076	0.077	0.078
DI[g]	0.0676	0.0712	0.0716	0.0721
QE[h]	1.0491	1.0558	1.0567	1.0574
σ_θ^2 [i]	140.40	160.10	162.81	164.91
ECoN[j]	5.034	4.969	4.962	4.955
*T*1-O2a	1.5894(6)	1.5885(4)	1.5891(4)	1.5891(4)
*T*1-O1a	1.6115(6)	1.6161(3)	1.6166(4)	1.6177(3)
*T*1-O3a	1.6457(6)	1.6387(4)	1.6385(4)	1.6390(4)
*T*1-O3a	1.6652(7)	1.6556(4)	1.6542(5)	1.6538(4)
<*T*1-O>	1.6280	1.6247	1.6246	1.6249
Poly. Vol.[e]	2.183	2.176	2.176	2.178
Poly. Vol. Dist.[f]	0.011	0.009	0.009	0.009
DI[g]	0.0169	0.0138	0.0134	0.0132
QE[h]	1.0098	1.0079	1.0076	1.0075
σ_θ^2 [i]	39.41	31.56	30.61	30.05
ECoN[j]	3.957	3.968	3.970	3.970
*T*2-O2b	1.5880(6)	1.5886(4)	1.5894(4)	1.5894(4)
*T*2-O1b	1.6192(6)	1.6233(3)	1.6238(4)	1.6251(3)
*T*2-O3b	1.6758(6)	1.6616(4)	1.6612(5)	1.6596(4)
*T*2-O3b	1.6774(7)	1.6639(4)	1.6624(4)	1.6618(4)

<*T*2-O>	1.6401	1.6344	1.6342	1.6340		
Poly. Vol.[e]	2.248	2.226	2.226	2.225		
Poly. Vol. Dist.[f]	0.006	0.005	0.005	0.005		
DI[g]	0.0223	0.0174	0.0169	0.0164		
QE[h]	1.0052	1.0047	1.0046	1.0046		
σ_θ^2 [i]	19.53	18.14	17.83	17.75		
ECoN[j]	3.928	3.952	3.954	3.956		
O3a-O3a-O3a	158.97(1)	161.26(1)	161.46(1)	161.68(1)		
O3b-O3b-O3b	139.06(1)	142.64(1)	143.08(1)	143.48(1)		
	4a	4d	protoenstatite[†]	Li-Sc-protopx[‡]	Li-Sc-protopx[‡‡]	$LiFeSi_2O_6$[#]
*M*1-O2 x2	1.9816(3)	1.9837(4)	1.984(4)	2.011(3)	2.007(4)	1.9117(11)
*M*1-O1 x2	2.0567(3)	2.0564(4)	2.064(4)	2.076(3)	2.087(4)	2.0291(12)
*M*1-O1 x2	2.1867(3)	2.1863(4)	2.220(4)	2.195(4)	2.202(4)	2.1333(11)
<*M*1-O>	2.0750	2.0754	2.089	2.094	2.099	2.0247
<i.r.> (Å)[d]	0.711	0.711	0.723	0.725	0.726	0.649
Poly. Vol.[e]	11.729	11.736	11.990	12.069	12.152	10.868
Poly. Vol. Dist.[f]	0.011	0.011	0.011	0.010	0.009	0.009
DI[g]	0.0359	0.0356	0.0417	0.0321	0.033	0.0372
QE[h]	1.0121	1.0120	1.0114	1.0108	1.011	1.0142
σ_θ^2 [i]	36.06	36.00	31.44	32.75	32.38	41.77
ECoN[j]	5.673	5.680	5.572	5.742	5.712	5.594
*M*2-O2 x2	2.0629(3)	2.0619(4)	2.057(4)	2.067(3)	2.080(4)	2.1805(13)
*M*2-O1 x2	2.0557(3)	2.0555(4)	2.047(4)	2.078(4)	2.090(4)	2.081(4)

*M*2-O3 x2	2.3458(3)	2.3465(5)	2.368(4)	2.373(4)	2.396(4)	2.488(4)
<*M*2-O2>	2.1548	2.1546	2.157	2.173	2.189	2.2497
<i.r.> (Å)[d]	0.738	0.737	0.723	0.743	0.750	0.812
Poly. Vol.[e]	11.112	11.110	11.305	11.335	11.528	11.241
Poly. Vol. Dist.[f]	0.173	0.173	0.161	0.176	0.181	0.070
DI[g]	0.0591	0.0594	0.0650	0.0616	0.063	0.0705
QE[h]	1.1341	1.1340	1.1248	1.1380	1.142	1.2290
σ_θ^2 [i]	328.38	328.11	307.14	332.52	338.29	480.27
ECoN[j]	5.279	5.273	5.142	5.223	5.184	4.922
T-O2	1.5893(3)	1.5887(4)	1.591(4)	1.585(4)	1.591(3)	1.5970(11)
T-O1	1.6140(3)	1.6148(3)	1.604(3)	1.613(3)	1.607(3)	1.6342(12)
T-O3	1.6414(3)	1.6414(4)	1.636(4)	1.637(3)	1.630(3)	1.6230(13)
T-O3	1.6560(3)	1.6559(4)	1.649(4)	1.661(3)	1.658(3)	1.6292(12)
<*T*-O>	1.6252	1.6252	1.620	1.624	1.622	1.6208
Poly. Vol.[e]	2.175	2.175	2.152	2.172	2.172	2.176
Poly. Vol. Dist.[f]	0.011	0.011	0.013	0.010	0.008	0.004
DI[g]	0.0145	0.0144	0.0139	0.0155	0.014	0.0074
QE[h]	1.0088	1.0088	1.0096	1.0085	1.007	1.0028
σ_θ^2 [i]	35.07	35.19	38.21	33.78	29.18	10.98
ECoN[j]	3.967	3.967	3.972	3.960	3.968	3.990
O3-O3-O3	164.71(1)	164.75(1)	163.09(1)	166.21(1)	167.31(1)	179.17(1)

Note: the reported parameters were calculated using VESTA 3 (Momma & Izumi, 2011) and ECoN21 (Ilinca, 2022).

[§] = Ganguly & Ghose (1979)

[†] Kanzaki & Xue (2017)

‡ Yang et al. (1999)

‡‡ Smyth & Ito (1977)

Redhammer & Roth (2004)

[d] mean ion radius (Hawthorne & Gagné, 2024)

[e] polyhedral volume ($Å^3$: Swanson & Peterson, 1980)

[f] polyhedron volume distortion (Makovicky & Balić-Žunić, 1998)

[g] distortion index (Baur, 1974)

[h] mean quadratic elongation (Robinson et al., 1971)

[i] bond angle variance (°^{2}) (Robinson et al., 1971)

[j] effective coordination number (Hoppe, 1979)

Table S4 Bond valence sum (in valence units vu) for the Li-Fe^{3+}-doped ortho (1, 2, 3) and protopyroxenes (4a and 4d). Data of orthoenstatite, protoenstatite, Li-Sc-protopyroxene and $LiFeSi_2O_6$ from literature are reported for comparison

	orthoenstatite§	1	2	3
*M*1	2.0980	2.3181	2.3390	2.3563
*M*2	1.8707	1.6149	1.5943	1.5707
*T*1	3.9708	4.0007	4.0019	3.9986
*T*2	3.8564	3.9071	3.9079	3.9103
O1a	2.0532	2.0338	2.0341	2.0296
O1b	2.0133	2.0025	2.0026	1.9992
O2a	1.8842	1.9067	1.9054	1.9065
O2b	1.8886	1.8849	1.8837	1.8829
O3a	2.0593	2.0799	2.0822	2.0802
O3b	1.8973	1.9331	1.9352	1.9374

	4a	4d	protoenstatite†	Li-Sc-protopx‡	Li-Sc-protopx‡‡	$LiFeSi_2O_6$#
*M*1	2.2078	2.2007	2.0741	2.2639	2.3142	3.0185
*M*2	1.7067	1.7136	1.8274	1.6042	1.5094	0.9763
T	3.9968	3.9963	4.0474	4.0091	4.0180	4.0357
O1	2.0194	2.0182	2.0240	2.0271	2.0330	2.0205
O2	1.8770	1.8773	1.8874	1.8741	1.8516	1.9143
O3	2.0576	2.0579	2.0876	2.0420	2.0452	2.0982

Note: Parameters were taken from Gagné & Hawthorne (2015). Bond valence was calculated at *M*1 and *M*2 in both the cases of the occupancy by Mg and Fe^{3+} and Mg and Li, respectively. For the oxygen sites, calculations were performed considering a correlated allocation of Mg at both *M*1 and *M*2, of Fe^{3+} and Li at *M*1 and *M*2, respectively, and the average value arising from the derived site populations at *M*1 and *M*2.

§ = Ganguly & Ghose (1979)

† Kanzaki & Xue (2017)

‡ Yang et al. (1999)

‡‡ Smyth & Ito (1977)

Redhammer & Roth (2004)

Figure S1 Dependence of volume from Li sof. The red and blue curves are guides to the eye showing the variation of Li in PPX and OPX, respectively.

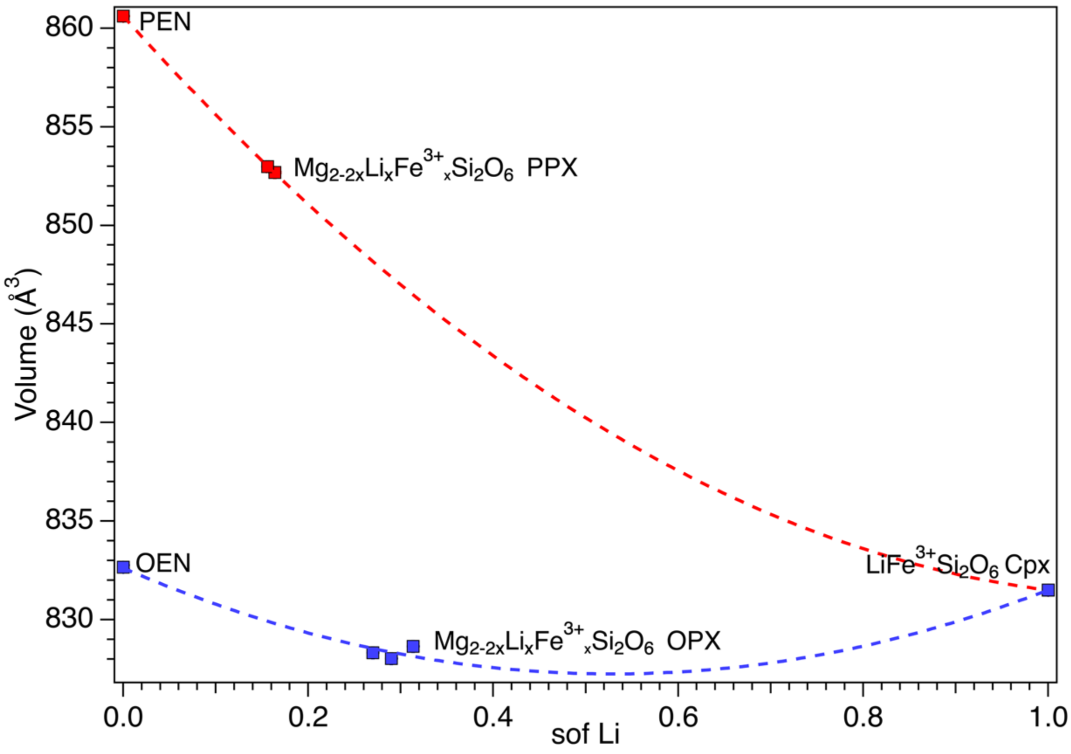

Figure S2 Dependence of the bond valence sum at O sites from Li sof. Solid curves are a guide to the eye. Coloured rectangular region highlights the range of the bond-valence sum at the O sites consistent with the bond-valence rule: 2.00 ± 0.05 vu

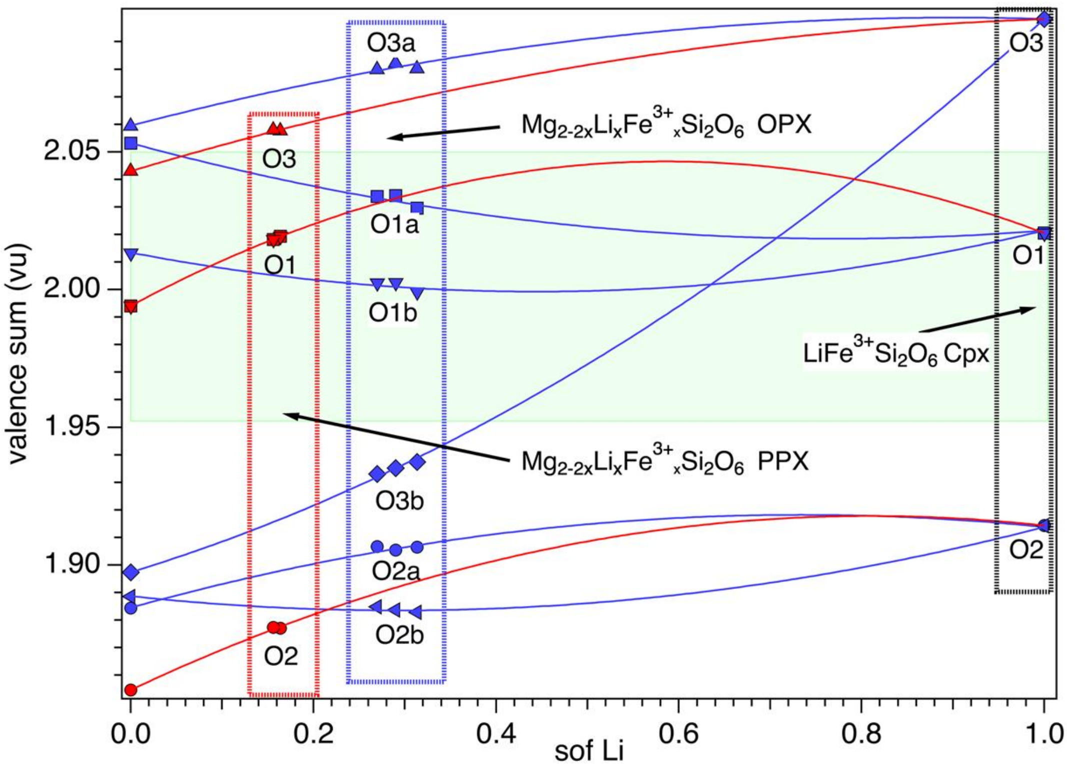

Supplement: Supplementary file 7 [file b-81-00084-sup7.pdf]
